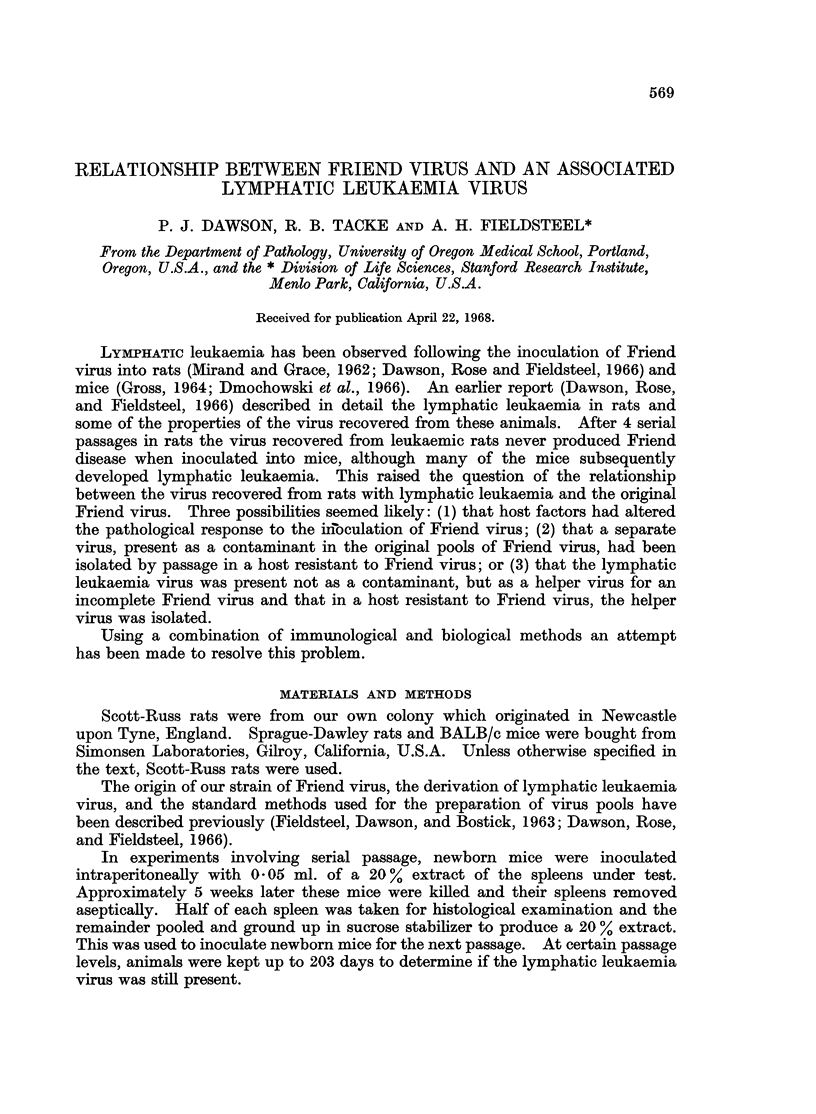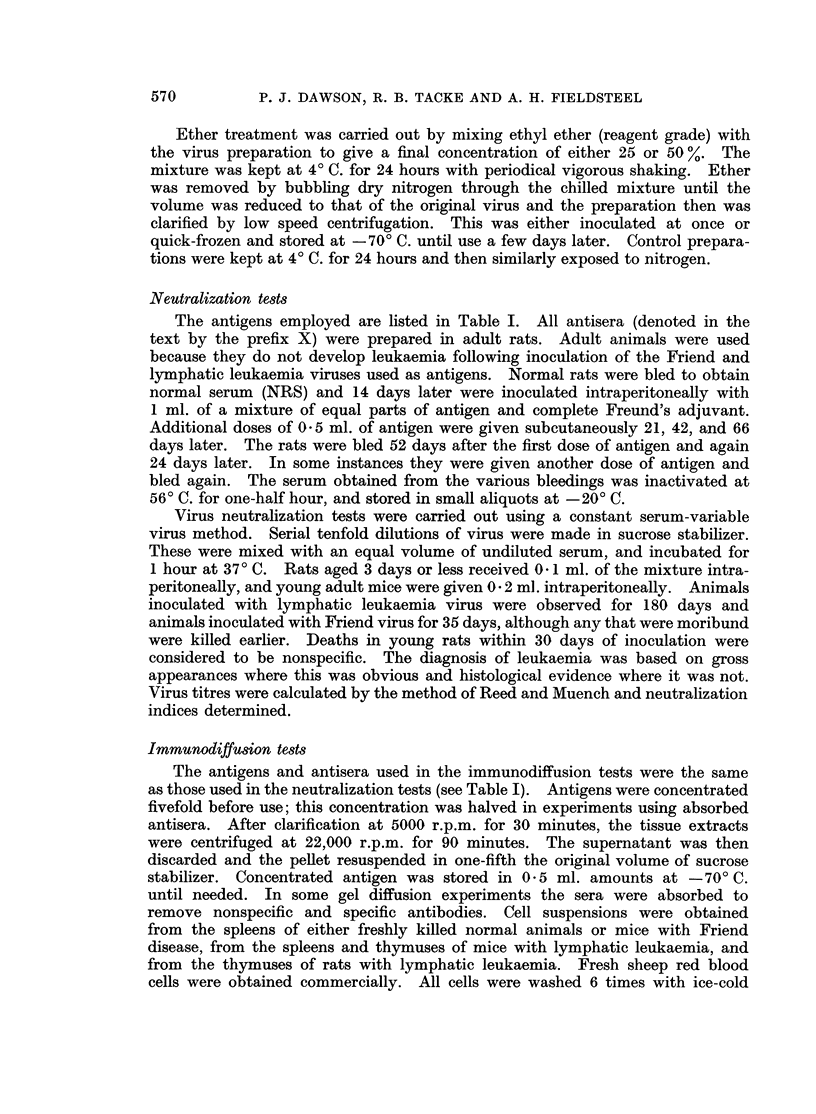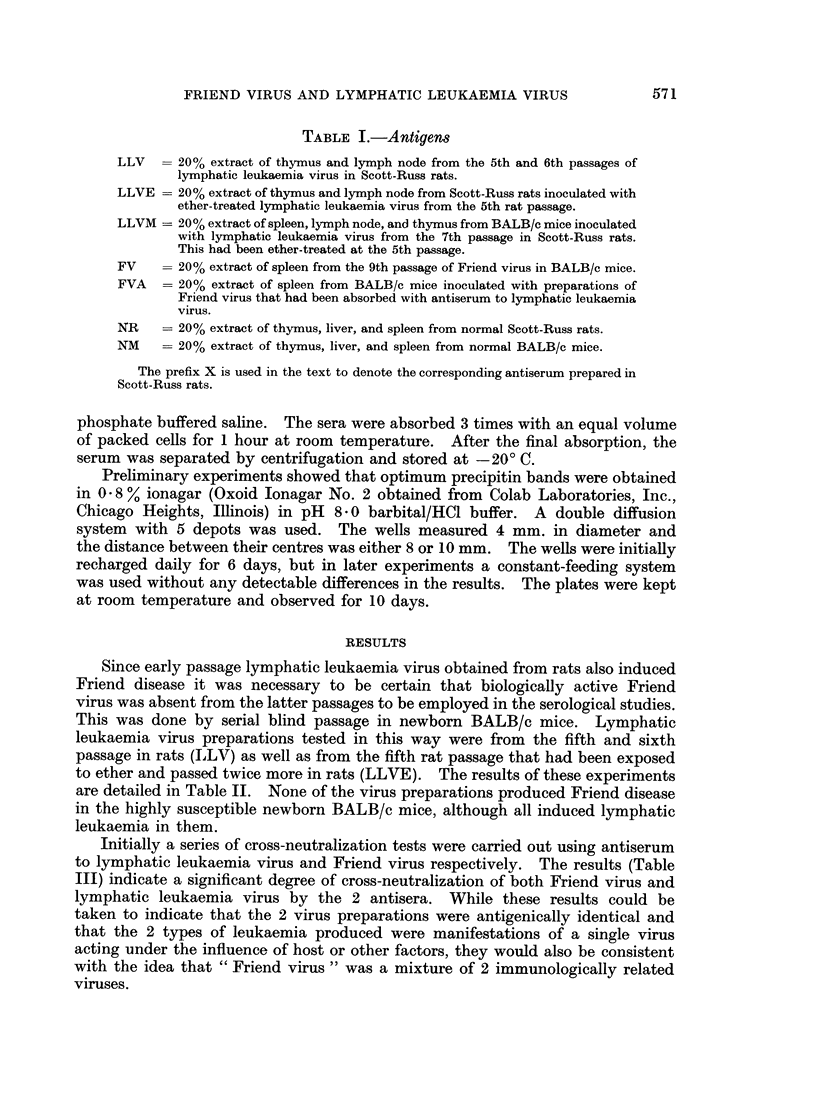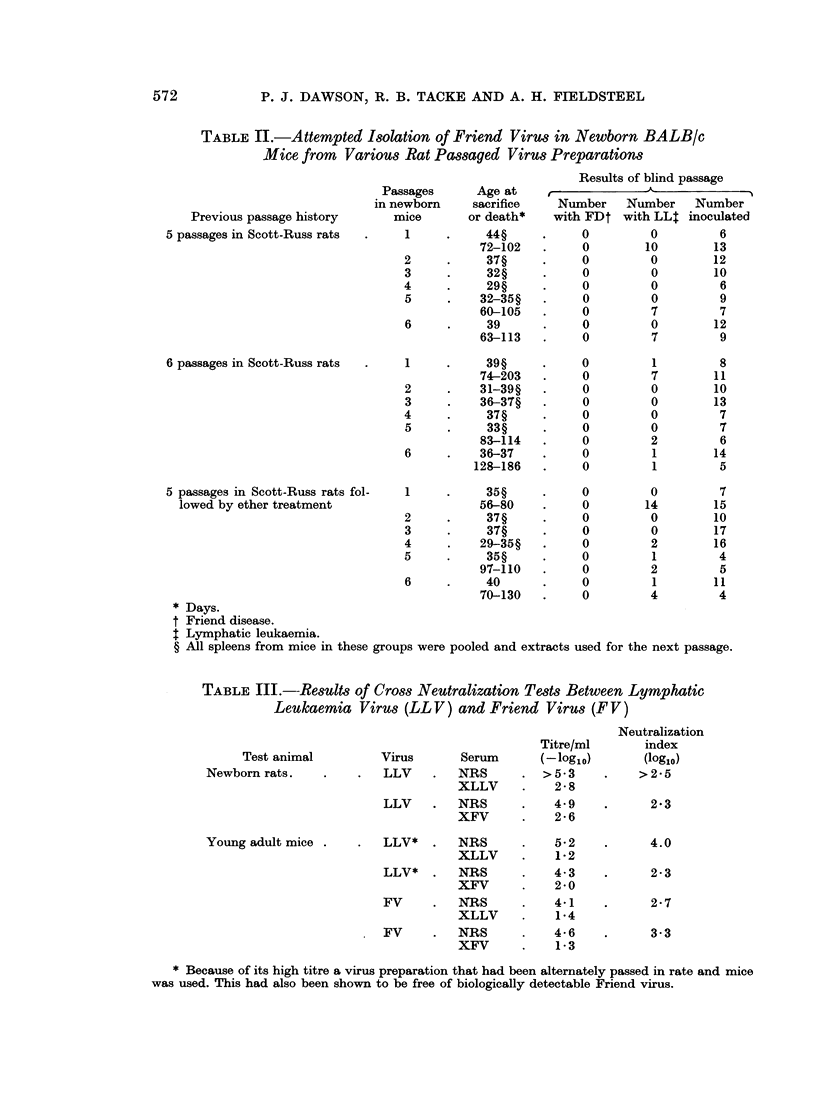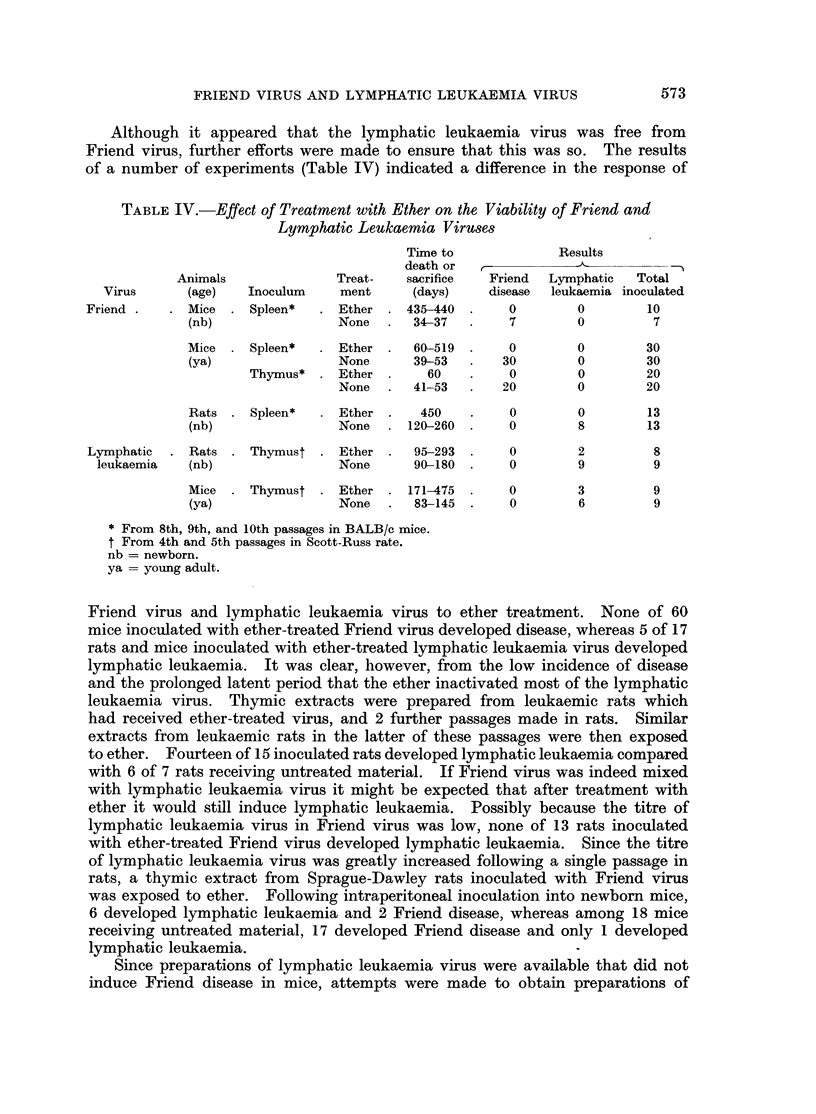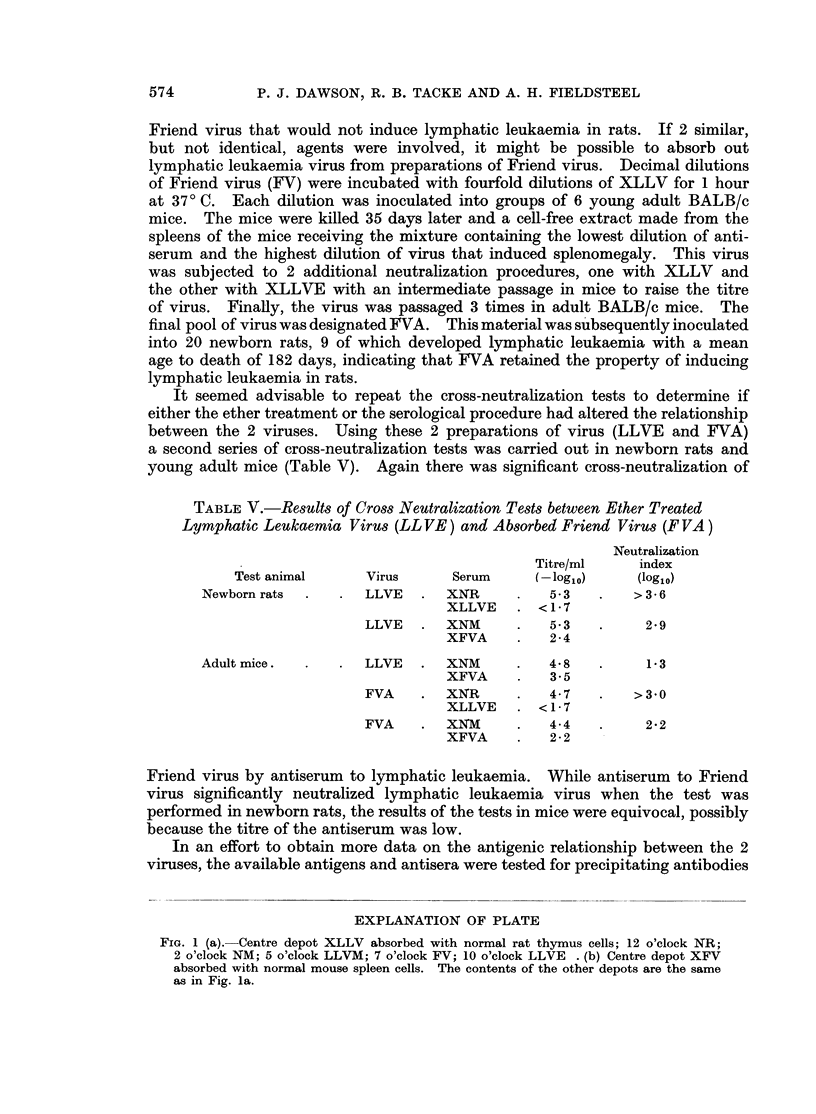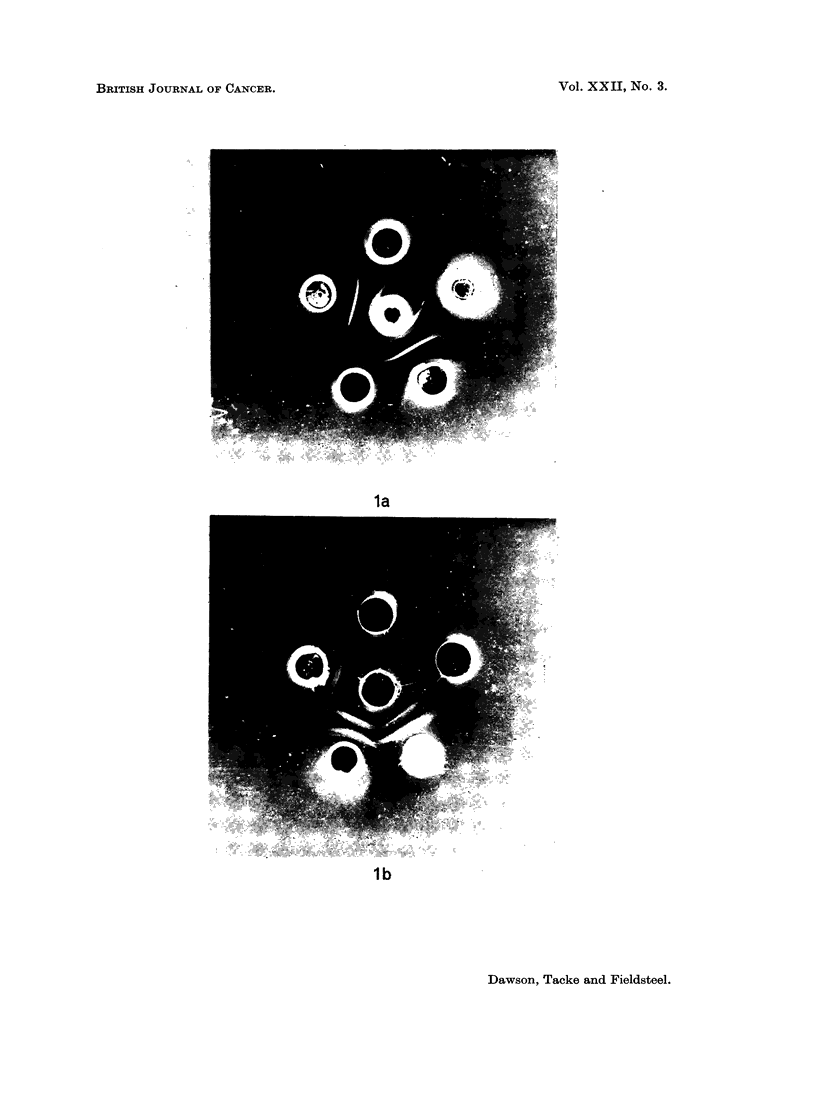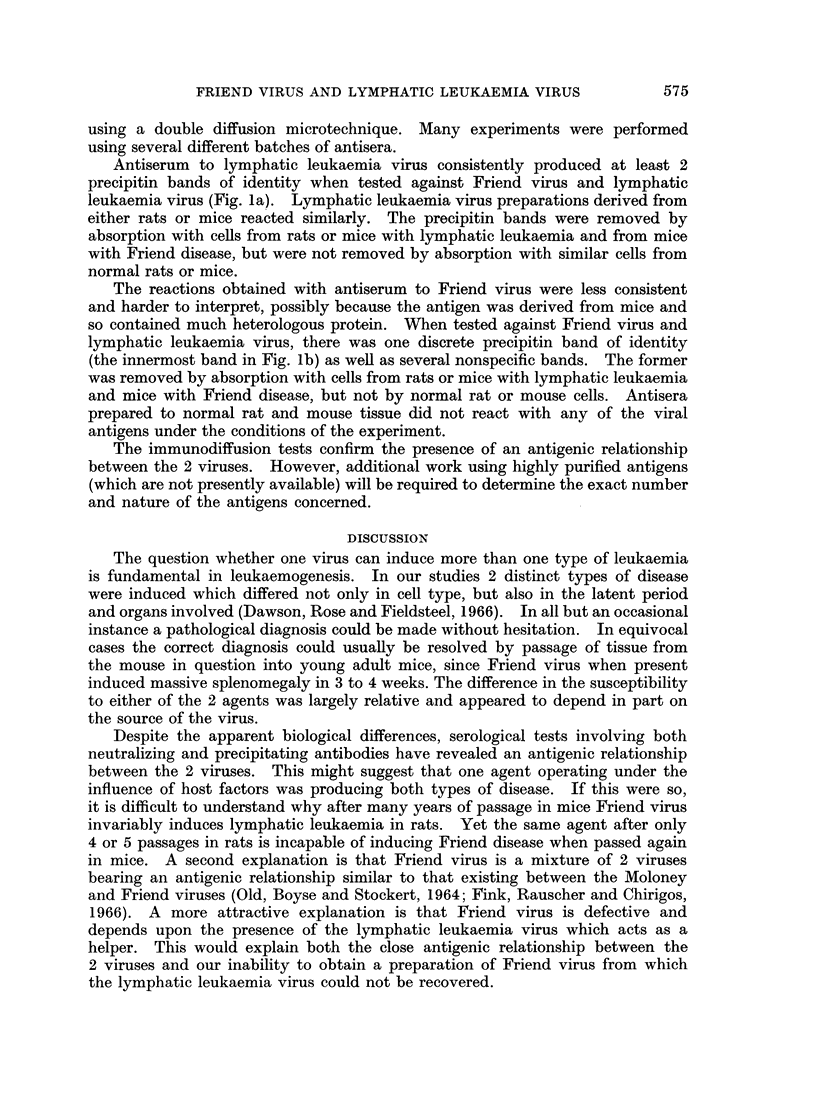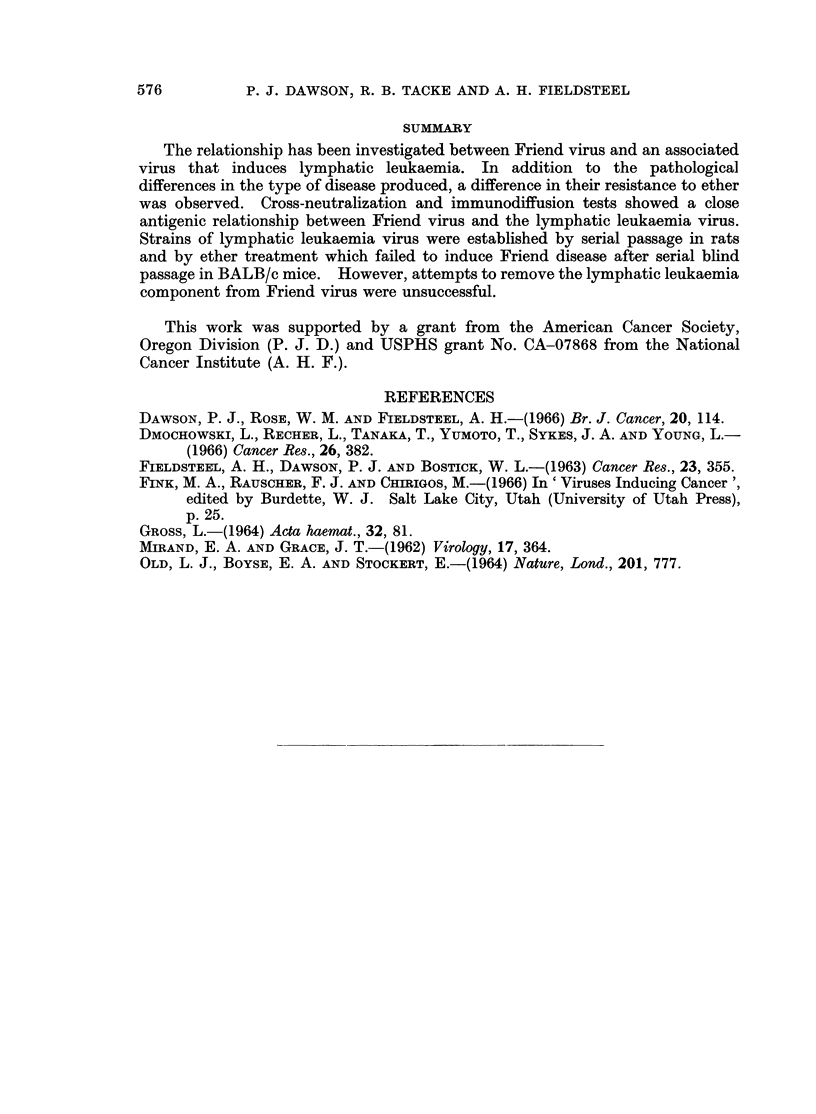# Relationship between Friend virus and an associated lymphatic leukaemia virus.

**DOI:** 10.1038/bjc.1968.67

**Published:** 1968-09

**Authors:** P. J. Dawson, R. B. Tacke, A. H. Fieldsteel

## Abstract

**Images:**


### RELATIONSHIP BETWEEN FRIEND VIRUS AND AN ASSOCIATED LYMPHATIC LEUKAEMIA VIRUS

P. J. DAWSON, R. B. TACKE AND A. H. FIELDSTEEL*

*From the Department of Pathology, University of Oregon Medical School, Portland, Oregon, U.S.A., and the * Division of Life Sciences, Stanford Research Institute, Menlo Park, California, U.S.A.*

Received for publication April 22, 1968.

LYMPHATIC leukaemia has been observed following the inoculation of Friend virus into rats (Mirand and Grace, 1962; Dawson, Rose and Fieldsteel, 1966) and mice (Gross, 1964; Dmochowski *et al.*, 1966). An earlier report (Dawson, Rose, and Fieldsteel, 1966) described in detail the lymphatic leukaemia in rats and some of the properties of the virus recovered from these animals. After 4 serial passages in rats the virus recovered from leukaemic rats never produced Friend disease when inoculated into mice, although many of the mice subsequently developed lymphatic leukaemia. This raised the question of the relationship between the virus recovered from rats with lymphatic leukaemia and the original Friend virus. Three possibilities seemed likely: (1) that host factors had altered the pathological response to the inoculation of Friend virus; (2) that a separate virus, present as a contaminant in the original pools of Friend virus, had been isolated by passage in a host resistant to Friend virus; or (3) that the lymphatic leukaemia virus was present not as a contaminant, but as a helper virus for an incomplete Friend virus and that in a host resistant to Friend virus, the helper virus was isolated.

Using a combination of immunological and biological methods an attempt has been made to resolve this problem.

### MATERIALS AND METHODS

Scott-Russ rats were from our own colony which originated in Newcastle upon Tyne, England. Sprague-Dawley rats and BALB/c mice were bought from Simonsen Laboratories, Gilroy, California, U.S.A. Unless otherwise specified in the text, Scott-Russ rats were used.

The origin of our strain of Friend virus, the derivation of lymphatic leukaemia virus, and the standard methods used for the preparation of virus pools have been described previously (Fieldsteel, Dawson, and Bostick, 1963; Dawson, Rose, and Fieldsteel, 1966).

In experiments involving serial passage, newborn mice were inoculated intraperitoneally with 0·05 ml. of a 20% extract of the spleens under test. Approximately 5 weeks later these mice were killed and their spleens removed aseptically. Half of each spleen was taken for histological examination and the remainder pooled and ground up in sucrose stabilizer to produce a 20% extract. This was used to inoculate newborn mice for the next passage. At certain passage levels, animals were kept up to 203 days to determine if the lymphatic leukaemia virus was still present.

Ether treatment was carried out by mixing ethyl ether (reagent grade) with the virus preparation to give a final concentration of either 25 or 50%. The mixture was kept at 4° C. for 24 hours with periodical vigorous shaking. Ether was removed by bubbling dry nitrogen through the chilled mixture until the volume was reduced to that of the original virus and the preparation then was clarified by low speed centrifugation. This was either inoculated at once or quick-frozen and stored at −70° C. until use a few days later. Control preparations were kept at 4° C. for 24 hours and then similarly exposed to nitrogen.

*Neutralization tests*

The antigens employed are listed in Table I. All antisera (denoted in the text by the prefix X) were prepared in adult rats. Adult animals were used because they do not develop leukaemia following inoculation of the Friend and lymphatic leukaemia viruses used as antigens. Normal rats were bled to obtain normal serum (NRS) and 14 days later were inoculated intraperitoneally with 1 ml. of a mixture of equal parts of antigen and complete Freund's adjuvant. Additional doses of 0·5 ml. of antigen were given subcutaneously 21, 42, and 66 days later. The rats were bled 52 days after the first dose of antigen and again 24 days later. In some instances they were given another dose of antigen and bled again. The serum obtained from the various bleedings was inactivated at 56° C. for one-half hour, and stored in small aliquots at −20° C.

Virus neutralization tests were carried out using a constant serum-variable virus method. Serial tenfold dilutions of virus were made in sucrose stabilizer. These were mixed with an equal volume of undiluted serum, and incubated for 1 hour at 37° C. Rats aged 3 days or less received 0·1 ml. of the mixture intraperitoneally, and young adult mice were given 0·2 ml. intraperitoneally. Animals inoculated with lymphatic leukaemia virus were observed for 180 days and animals inoculated with Friend virus for 35 days, although any that were moribund were killed earlier. Deaths in young rats within 30 days of inoculation were considered to be nonspecific. The diagnosis of leukaemia was based on gross appearances where this was obvious and histological evidence where it was not. Virus titres were calculated by the method of Reed and Muench and neutralization indices determined.

*Immunodiffusion tests*

The antigens and antisera used in the immunodiffusion tests were the same as those used in the neutralization tests (see Table I). Antigens were concentrated fivefold before use; this concentration was halved in experiments using absorbed antisera. After clarification at 5000 r.p.m. for 30 minutes, the tissue extracts were centrifuged at 22,000 r.p.m. for 90 minutes. The supernatant was then discarded and the pellet resuspended in one-fifth the original volume of sucrose stabilizer. Concentrated antigen was stored in 0·5 ml. amounts at −70° C. until needed. In some gel diffusion experiments the sera were absorbed to remove nonspecific and specific antibodies. Cell suspensions were obtained from the spleens of either freshly killed normal animals or mice with Friend disease, from the spleens and thymuses of mice with lymphatic leukaemia, and from the thymuses of rats with lymphatic leukaemia. Fresh sheep red blood cells were obtained commercially. All cells were washed 6 times with ice-cold

TABLE I.—*Antigens*

| | |
|---|---|
| LLV | = 20% extract of thymus and lymph node from the 5th and 6th passages of lymphatic leukaemia virus in Scott-Russ rats. |
| LLVE | = 20% extract of thymus and lymph node from Scott-Russ rats inoculated with ether-treated lymphatic leukaemia virus from the 5th rat passage. |
| LLVM | = 20% extract of spleen, lymph node, and thymus from BALB/c mice inoculated with lymphatic leukaemia virus from the 7th passage in Scott-Russ rats. This had been ether-treated at the 5th passage. |
| FV | = 20% extract of spleen from the 9th passage of Friend virus in BALB/c mice. |
| FVA | = 20% extract of spleen from BALB/c mice inoculated with preparations of Friend virus that had been absorbed with antiserum to lymphatic leukaemia virus. |
| NR | = 20% extract of thymus, liver, and spleen from normal Scott-Russ rats. |
| NM | = 20% extract of thymus, liver, and spleen from normal BALB/c mice. |

The prefix X is used in the text to denote the corresponding antiserum prepared in Scott-Russ rats.

phosphate buffered saline. The sera were absorbed 3 times with an equal volume of packed cells for 1 hour at room temperature. After the final absorption, the serum was separated by centrifugation and stored at −20° C.

Preliminary experiments showed that optimum precipitin bands were obtained in 0·8% ionagar (Oxoid Ionagar No. 2 obtained from Colab Laboratories, Inc., Chicago Heights, Illinois) in pH 8·0 barbital/HCl buffer. A double diffusion system with 5 depots was used. The wells measured 4 mm. in diameter and the distance between their centres was either 8 or 10 mm. The wells were initially recharged daily for 6 days, but in later experiments a constant-feeding system was used without any detectable differences in the results. The plates were kept at room temperature and observed for 10 days.

### RESULTS

Since early passage lymphatic leukaemia virus obtained from rats also induced Friend disease it was necessary to be certain that biologically active Friend virus was absent from the latter passages to be employed in the serological studies. This was done by serial blind passage in newborn BALB/c mice. Lymphatic leukaemia virus preparations tested in this way were from the fifth and sixth passage in rats (LLV) as well as from the fifth rat passage that had been exposed to ether and passed twice more in rats (LLVE). The results of these experiments are detailed in Table II. None of the virus preparations produced Friend disease in the highly susceptible newborn BALB/c mice, although all induced lymphatic leukaemia in them.

Initially a series of cross-neutralization tests were carried out using antiserum to lymphatic leukaemia virus and Friend virus respectively. The results (Table III) indicate a significant degree of cross-neutralization of both Friend virus and lymphatic leukaemia virus by the 2 antisera. While these results could be taken to indicate that the 2 virus preparations were antigenically identical and that the 2 types of leukaemia produced were manifestations of a single virus acting under the influence of host or other factors, they would also be consistent with the idea that " Friend virus " was a mixture of 2 immunologically related viruses.

TABLE II.—*Attempted Isolation of Friend Virus in Newborn BALB/c Mice from Various Rat Passaged Virus Preparations*

| Previous passage history | Passages in newborn mice | Age at sacrifice or death* | Results of blind passage: Number with FD† | Number with LL‡ | Number inoculated |
|---|---|---|---|---|---|
| 5 passages in Scott-Russ rats | 1 | 44§ | 0 | 0 | 6 |
| | | 72–102 | 0 | 10 | 13 |
| | 2 | 37§ | 0 | 0 | 12 |
| | 3 | 32§ | 0 | 0 | 10 |
| | 4 | 29§ | 0 | 0 | 6 |
| | 5 | 32–35§ | 0 | 0 | 9 |
| | | 60–105 | 0 | 7 | 7 |
| | 6 | 39 | 0 | 0 | 12 |
| | | 63–113 | 0 | 7 | 9 |
| 6 passages in Scott-Russ rats | 1 | 39§ | 0 | 1 | 8 |
| | | 74–203 | 0 | 7 | 11 |
| | 2 | 31–39§ | 0 | 0 | 10 |
| | 3 | 36–37§ | 0 | 0 | 13 |
| | 4 | 37§ | 0 | 0 | 7 |
| | 5 | 33§ | 0 | 0 | 7 |
| | | 83–114 | 0 | 2 | 6 |
| | 6 | 36–37 | 0 | 1 | 14 |
| | | 128–186 | 0 | 1 | 5 |
| 5 passages in Scott-Russ rats followed by ether treatment | 1 | 35§ | 0 | 0 | 7 |
| | | 56–80 | 0 | 14 | 15 |
| | 2 | 37§ | 0 | 0 | 10 |
| | 3 | 37§ | 0 | 0 | 17 |
| | 4 | 29–35§ | 0 | 2 | 16 |
| | 5 | 35§ | 0 | 1 | 4 |
| | | 97–110 | 0 | 2 | 5 |
| | 6 | 40 | 0 | 1 | 11 |
| | | 70–130 | 0 | 4 | 4 |

* Days.
† Friend disease.
‡ Lymphatic leukaemia.
§ All spleens from mice in these groups were pooled and extracts used for the next passage.

TABLE III.—*Results of Cross Neutralization Tests Between Lymphatic Leukaemia Virus (LLV) and Friend Virus (FV)*

| Test animal | Virus | Serum | Titre/ml ($-\log_{10}$) | Neutralization index ($\log_{10}$) |
|---|---|---|---|---|
| Newborn rats | LLV | NRS | >5·3 | >2·5 |
| | | XLLV | 2·8 | |
| | LLV | NRS | 4·9 | 2·3 |
| | | XFV | 2·6 | |
| Young adult mice | LLV* | NRS | 5·2 | 4.0 |
| | | XLLV | 1·2 | |
| | LLV* | NRS | 4·3 | 2·3 |
| | | XFV | 2·0 | |
| | FV | NRS | 4·1 | 2·7 |
| | | XLLV | 1·4 | |
| | FV | NRS | 4·6 | 3·3 |
| | | XFV | 1·3 | |

* Because of its high titre a virus preparation that had been alternately passed in rate and mice was used. This had also been shown to be free of biologically detectable Friend virus.

Although it appeared that the lymphatic leukaemia virus was free from Friend virus, further efforts were made to ensure that this was so. The results of a number of experiments (Table IV) indicated a difference in the response of

TABLE IV.—*Effect of Treatment with Ether on the Viability of Friend and Lymphatic Leukaemia Viruses*

| Virus | Animals (age) | Inoculum | Treatment | Time to death or sacrifice (days) | Results: Friend disease | Results: Lymphatic leukaemia | Results: Total inoculated |
|---|---|---|---|---|---|---|---|
| Friend | Mice (nb) | Spleen* | Ether | 435–440 | 0 | 0 | 10 |
| | | | None | 34–37 | 7 | 0 | 7 |
| | Mice (ya) | Spleen* | Ether | 60–519 | 0 | 0 | 30 |
| | | | None | 39–53 | 30 | 0 | 30 |
| | | Thymus* | Ether | 60 | 0 | 0 | 20 |
| | | | None | 41–53 | 20 | 0 | 20 |
| | Rats (nb) | Spleen* | Ether | 450 | 0 | 0 | 13 |
| | | | None | 120–260 | 0 | 8 | 13 |
| Lymphatic leukaemia | Rats (nb) | Thymus† | Ether | 95–293 | 0 | 2 | 8 |
| | | | None | 90–180 | 0 | 9 | 9 |
| | Mice (ya) | Thymus† | Ether | 171–475 | 0 | 3 | 9 |
| | | | None | 83–145 | 0 | 6 | 9 |

* From 8th, 9th, and 10th passages in BALB/c mice.
† From 4th and 5th passages in Scott-Russ rate.
nb = newborn.
ya = young adult.

Friend virus and lymphatic leukaemia virus to ether treatment. None of 60 mice inoculated with ether-treated Friend virus developed disease, whereas 5 of 17 rats and mice inoculated with ether-treated lymphatic leukaemia virus developed lymphatic leukaemia. It was clear, however, from the low incidence of disease and the prolonged latent period that the ether inactivated most of the lymphatic leukaemia virus. Thymic extracts were prepared from leukaemic rats which had received ether-treated virus, and 2 further passages made in rats. Similar extracts from leukaemic rats in the latter of these passages were then exposed to ether. Fourteen of 15 inoculated rats developed lymphatic leukaemia compared with 6 of 7 rats receiving untreated material. If Friend virus was indeed mixed with lymphatic leukaemia virus it might be expected that after treatment with ether it would still induce lymphatic leukaemia. Possibly because the titre of lymphatic leukaemia virus in Friend virus was low, none of 13 rats inoculated with ether-treated Friend virus developed lymphatic leukaemia. Since the titre of lymphatic leukaemia virus was greatly increased following a single passage in rats, a thymic extract from Sprague-Dawley rats inoculated with Friend virus was exposed to ether. Following intraperitoneal inoculation into newborn mice, 6 developed lymphatic leukaemia and 2 Friend disease, whereas among 18 mice receiving untreated material, 17 developed Friend disease and only 1 developed lymphatic leukaemia.

Since preparations of lymphatic leukaemia virus were available that did not induce Friend disease in mice, attempts were made to obtain preparations of

Friend virus that would not induce lymphatic leukaemia in rats. If 2 similar, but not identical, agents were involved, it might be possible to absorb out lymphatic leukaemia virus from preparations of Friend virus. Decimal dilutions of Friend virus (FV) were incubated with fourfold dilutions of XLLV for 1 hour at 37° C. Each dilution was inoculated into groups of 6 young adult BALB/c mice. The mice were killed 35 days later and a cell-free extract made from the spleens of the mice receiving the mixture containing the lowest dilution of antiserum and the highest dilution of virus that induced splenomegaly. This virus was subjected to 2 additional neutralization procedures, one with XLLV and the other with XLLVE with an intermediate passage in mice to raise the titre of virus. Finally, the virus was passaged 3 times in adult BALB/c mice. The final pool of virus was designated FVA. This material was subsequently inoculated into 20 newborn rats, 9 of which developed lymphatic leukaemia with a mean age to death of 182 days, indicating that FVA retained the property of inducing lymphatic leukaemia in rats.

It seemed advisable to repeat the cross-neutralization tests to determine if either the ether treatment or the serological procedure had altered the relationship between the 2 viruses. Using these 2 preparations of virus (LLVE and FVA) a second series of cross-neutralization tests was carried out in newborn rats and young adult mice (Table V). Again there was significant cross-neutralization of

TABLE V.—*Results of Cross Neutralization Tests between Ether Treated Lymphatic Leukaemia Virus (LLVE) and Absorbed Friend Virus (FVA)*

| Test animal | Virus | Serum | Titre/ml ($-\log_{10}$) | Neutralization index ($\log_{10}$) |
|---|---|---|---|---|
| Newborn rats | LLVE | XNR | 5·3 | >3·6 |
| | | XLLVE | <1·7 | |
| | LLVE | XNM | 5·3 | 2·9 |
| | | XFVA | 2·4 | |
| Adult mice | LLVE | XNM | 4·8 | 1·3 |
| | | XFVA | 3·5 | |
| | FVA | XNR | 4·7 | >3·0 |
| | | XLLVE | <1·7 | |
| | FVA | XNM | 4·4 | 2·2 |
| | | XFVA | 2·2 | |

Friend virus by antiserum to lymphatic leukaemia. While antiserum to Friend virus significantly neutralized lymphatic leukaemia virus when the test was performed in newborn rats, the results of the tests in mice were equivocal, possibly because the titre of the antiserum was low.

In an effort to obtain more data on the antigenic relationship between the 2 viruses, the available antigens and antisera were tested for precipitating antibodies

EXPLANATION OF PLATE

FIG. 1 (a).—Centre depot XLLV absorbed with normal rat thymus cells; 12 o'clock NR; 2 o'clock NM; 5 o'clock LLVM; 7 o'clock FV; 10 o'clock LLVE . (b) Centre depot XFV absorbed with normal mouse spleen cells. The contents of the other depots are the same as in Fig. 1a.

1a

1b

Dawson, Tacke and Fieldsteel.

using a double diffusion microtechnique. Many experiments were performed using several different batches of antisera.

Antiserum to lymphatic leukaemia virus consistently produced at least 2 precipitin bands of identity when tested against Friend virus and lymphatic leukaemia virus (Fig. 1a). Lymphatic leukaemia virus preparations derived from either rats or mice reacted similarly. The precipitin bands were removed by absorption with cells from rats or mice with lymphatic leukaemia and from mice with Friend disease, but were not removed by absorption with similar cells from normal rats or mice.

The reactions obtained with antiserum to Friend virus were less consistent and harder to interpret, possibly because the antigen was derived from mice and so contained much heterologous protein. When tested against Friend virus and lymphatic leukaemia virus, there was one discrete precipitin band of identity (the innermost band in Fig. 1b) as well as several nonspecific bands. The former was removed by absorption with cells from rats or mice with lymphatic leukaemia and mice with Friend disease, but not by normal rat or mouse cells. Antisera prepared to normal rat and mouse tissue did not react with any of the viral antigens under the conditions of the experiment.

The immunodiffusion tests confirm the presence of an antigenic relationship between the 2 viruses. However, additional work using highly purified antigens (which are not presently available) will be required to determine the exact number and nature of the antigens concerned.

### DISCUSSION

The question whether one virus can induce more than one type of leukaemia is fundamental in leukaemogenesis. In our studies 2 distinct types of disease were induced which differed not only in cell type, but also in the latent period and organs involved (Dawson, Rose and Fieldsteel, 1966). In all but an occasional instance a pathological diagnosis could be made without hesitation. In equivocal cases the correct diagnosis could usually be resolved by passage of tissue from the mouse in question into young adult mice, since Friend virus when present induced massive splenomegaly in 3 to 4 weeks. The difference in the susceptibility to either of the 2 agents was largely relative and appeared to depend in part on the source of the virus.

Despite the apparent biological differences, serological tests involving both neutralizing and precipitating antibodies have revealed an antigenic relationship between the 2 viruses. This might suggest that one agent operating under the influence of host factors was producing both types of disease. If this were so, it is difficult to understand why after many years of passage in mice Friend virus invariably induces lymphatic leukaemia in rats. Yet the same agent after only 4 or 5 passages in rats is incapable of inducing Friend disease when passed again in mice. A second explanation is that Friend virus is a mixture of 2 viruses bearing an antigenic relationship similar to that existing between the Moloney and Friend viruses (Old, Boyse and Stockert, 1964; Fink, Rauscher and Chirigos, 1966). A more attractive explanation is that Friend virus is defective and depends upon the presence of the lymphatic leukaemia virus which acts as a helper. This would explain both the close antigenic relationship between the 2 viruses and our inability to obtain a preparation of Friend virus from which the lymphatic leukaemia virus could not be recovered.

SUMMARY

The relationship has been investigated between Friend virus and an associated virus that induces lymphatic leukaemia. In addition to the pathological differences in the type of disease produced, a difference in their resistance to ether was observed. Cross-neutralization and immunodiffusion tests showed a close antigenic relationship between Friend virus and the lymphatic leukaemia virus. Strains of lymphatic leukaemia virus were established by serial passage in rats and by ether treatment which failed to induce Friend disease after serial blind passage in BALB/c mice. However, attempts to remove the lymphatic leukaemia component from Friend virus were unsuccessful.

This work was supported by a grant from the American Cancer Society, Oregon Division (P. J. D.) and USPHS grant No. CA–07868 from the National Cancer Institute (A. H. F.).

### REFERENCES

Dawson, P. J., Rose, W. M. and Fieldsteel, A. H.—(1966) *Br. J. Cancer*, **20**, 114.

Dmochowski, L., Recher, L., Tanaka, T., Yumoto, T., Sykes, J. A. and Young, L.—(1966) *Cancer Res.*, **26**, 382.

Fieldsteel, A. H., Dawson, P. J. and Bostick, W. L.—(1963) *Cancer Res.*, **23**, 355.

Fink, M. A., Rauscher, F. J. and Chirigos, M.—(1966) In ' Viruses Inducing Cancer ', edited by Burdette, W. J. Salt Lake City, Utah (University of Utah Press), p. 25.

Gross, L.—(1964) *Acta haemat.*, **32**, 81.

Mirand, E. A. and Grace, J. T.—(1962) *Virology*, **17**, 364.

Old, L. J., Boyse, E. A. and Stockert, E.—(1964) *Nature, Lond.*, **201**, 777.